# Assessment of Efficacy of a Novel Crosslinking Protocol with Intracameral Oxygen (Bubble-CXL) in Increasing the Corneal Stiffness Using Atomic Force Microscopy

**DOI:** 10.3390/nano12183185

**Published:** 2022-09-14

**Authors:** Ammar Alkhalde, Hannah Seferovic, Ali Abri, Alvana Simbrunner, Peter Hinterdorfer, Yoo Jin Oh

**Affiliations:** 1 Department of Ophthalmology, Hospital Wels-Grieskirchen, 4600 Wels, Austria; 2 Institute of Biophysics, Johannes Kepler University, 4020 Linz, Austria

**Keywords:** cornea, corneal crosslinking, intracameral oxygen, atomic force microscopy, force spectroscopy, mechanical mapping, biomechanics

## Abstract

The environmental oxygen level plays a critical role in corneal crosslinking (CXL), a treatment method to increase corneal biomechanical stability. In this study, we introduce a new CXL method (Bubble-CXL), in which intracameral oxygen serves as an additional oxygen source during eye treatment. The efficiency of this new method was compared with the efficiency of the standard CXL method. Three fresh porcine eye pairs were included in this study. One eye of each pair was treated with standard CXL, whereas in the partner eye, intracameral oxygen was injected prior to CXL and removed at the end of the procedure. The Young’s modulus of each cornea was measured using atomic force microscopy. All analyzed corneas treated with intracameral oxygen showed significantly higher Young’s modulus and thus an increased stiffness compared to the cornea of the partner eye treated with the standard protocol. Using intracameral oxygen in CXL therapy may increase crosslinking efficiency and improve biomechanical corneal properties.

## 1. Introduction

Keratoconus is a progressive bilateral and non-inflammatory corneal disorder, which leads to biomechanical instability of the cornea and hence to corneal thinning and steepening as well as visual deterioration [1,2,3]. A treatment method for preventing keratoconus progression is corneal crosslinking (CXL), which is based on bridging collagen fibrils with riboflavin and ultraviolet A (UVA) light [4]. Riboflavin is a photosensitizer that can diffuse into the corneal stroma in the absence of the epithelium [5] and generates singlet oxygen when excited with UVA radiation [4,6]. The singlet oxygen induces collagen crosslinking by forming covalent bonds bridging the amino groups of collagen fibrils [4].

The oxygen level during corneal crosslinking plays a critical role in effective treatment. Richoz et al. showed that corneas cross-linked under normal oxygen levels had a significant increase in Young’s modulus. In contrast, no effect was observed for corneas treated in a low oxygen environment [7]. Furthermore, Wang et al. observed a higher cross-linking efficiency for corneas when kept in a high oxygen environment during UVA irradiation [8]. Therefore, an additional external oxygen source seems to be a promising tool for enhancing the effect of CXL. So far, different devices have been developed to increase the oxygen concentration in the environment above the cornea. Some of them use components that are in contact with the patient during treatment [9,10]. Recently, a non-contact oxygen delivery device has been introduced, with which 99% oxygen concentration can be reached when brought in proximity of 8 to 10 mm to the treated cornea [11].

In this study, we introduce a novel CXL protocol (Bubble-CXL), in which intracameral oxygen is used for oxygen enrichment in the stroma in addition to the outer air environment. For a first assessment of the method’s efficiency, Young’s modulus was derived for corneas treated with conventional CXL (Dresden protocol) or Bubble-CXL, providing a quantitative comparison between the two methods and, thus, examining the impact of oxygen enrichment in the eye’s anterior chamber.

## 2. Materials and Methods

### 2.1. Corneal Crosslinking

Corneal crosslinking was performed on three fresh porcine pairs of eyes, which were stored in cold water before treatment. One eye of each pair was treated with the conventional CXL method (Dresden protocol) [4,12,13], and the other one with the Bubble-CXL method. The epithelium was removed for eyes treated with conventional CXL, and the cornea was instilled with 0.1% riboflavin solution (MedioCROSS^®^, formulation: 0.1% riboflavin, 20% Dextran) every 5 min for 30 min. Next, the cornea was irradiated for 30 min with ultraviolet A light (with an irradiance of 3 mW∙cm^−2^ and a wavelength of 370 nm). Meanwhile, the instillation of 0.1% riboflavin every 5 min was continued. The epithelium was removed for eyes treated with Bubble-CXL, and intracameral oxygen was injected until 80% of the anterior chamber was filled. Next, 0.1% riboflavin solution was instilled every 5 min for 30 min. The cornea was irradiated with ultraviolet A light (with an irradiance of 3 mW∙cm^−2^ and a wavelength of 370 nm) for 30 min, while 0.1% riboflavin solution was continuously instilled every 5 min. After CXL treatment, the intracameral oxygen was removed. For oxygen injection and removal, a Sauter air injection cannula (27 gauge) was used. A whole central corneal punch of 6 mm diameter and an average height of 0.87 ± 0.07 mm was extracted after crosslinking and fixed on a glass slide with epoxy glue. The sample was stored in 0.9% NaCl solution at a temperature of 4 °C. Stiffness measurements were performed on the day after CXL treatment.

### 2.2. Young’s Modulus Assessment

Silicon probes with a nominal spring constant of 2.8 N·m^−1^ (PPP-FM, Nanosensors, Neuchatel, Switzerland), similar to spring constants used in related studies [14,15,16], were used for the indentation experiments performed with atomic force microscopy (AFM). Before performing indentation experiments, the cantilever sensitivity (proportionality between the output voltage of the photodetector and cantilever deflection) was derived from the slope of a force–distance curve recorded on a rigid glass substrate. The exact spring constant of each cantilever was determined with the thermal noise method [17]. Measurements were performed in PBS (phosphate buffer saline, pH 7.4). The samples were covered with buffer during the entire measurement process to prevent them from drying. No damage to the sample or a noticeable temperature increase inside the measurement chamber due to the AFM laser was observed. Force–distance curves were recorded in the force volume mode on an 8 × 8 grid with a 4 μm^2^ area, with an approach and retraction speed of 2 μm·s^−1^ and a maximum indentation force of 10 nN. Such force volume measurements were recorded at up to 12 different positions on each cornea sample. Indentation measurements on the cornea of partner eyes were performed with the same cantilever and settings. Corneas of three pairs of eyes were characterized. Young’s modulus calculation was performed according to the Hertz model [18,19] with the software PicoView 1.20 (Keysight Technologies, Santa Clara, CA, USA). In the Hertz model, Young’s modulus E is directly proportional to the force F of the indenting AFM tip acting on the sample surface and inversely proportional to the 3/2 power of the deformation δ,

(1) $$F\;=\frac{4}{3}\frac{E}{\left(1-\nu^{2}\right)}\sqrt{\delta^{3}R}\;,$$

where R is the radius of the AFM tip and ν is the Poisson ratio of the elastic surface [18,19,20]. Each recorded force–distance curve was transformed into a force–indentation (F–δ) curve by subtracting the cantilever deflection on the cornea sample from the cantilever deflection on a hard glass substrate. In the next step, an algorithm described by Fuhrmann et al. [16] was used. The F–δ curve was transformed into an F^2/3^–δ curve to directly derive Young’s modulus E from the slope of the curve by applying a linear fit [20]:

(2) $$\frac{{\Delta F}^{2/3}}{\Delta \delta }={\left(\;\frac{4}{3}\frac{E}{\left(1-\nu^{2}\right)}\sqrt{R}\right)}^{2/3}\;$$

A tip radius R of 10 nm and Poisson’s ratio ν of 0.5 for biological samples were used for calculations.

### 2.3. Statistical Analysis

Statistical analysis was performed with the OriginPro 2019 software (9.60, OriginLab Corporation, Northampton, MA, USA). The Young’s modulus distributions for each pair of eyes were tested for normal distribution (Shapiro–Wilk test) and the equality of variances. As no normal distribution was found and variances differed significantly, the Mann–Whitney U test was performed to derive significant differences between Young’s modulus distributions of corneas treated with conventional CXL versus corneas treated with Bubble-CXL.

## 3. Results

**Figure 1:**
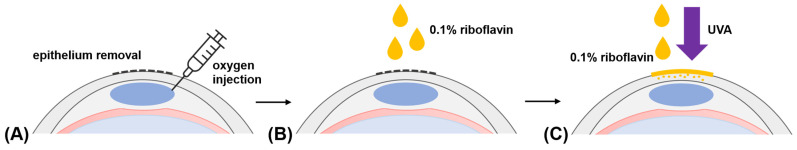
Bubble-CXL: (**A**) The epithelium is removed, and intracameral oxygen is injected into the eye. (**B**) A 0.1% riboflavin solution is instilled on the eye every 5 min for 30 min, (**C**) followed by irradiation with UVA (irradiance of 3 mW·cm^−2^ and a wavelength of 370 nm), while instillation with 0.1% riboflavin every 5 min is continued. After treatment, oxygen is removed from the anterior chamber.

A new protocol for corneal crosslinking (Bubble-CXL) was developed, and the efficiency of the method was tested by comparing Young’s modulus of corneas treated with conventional CXL (Dresden protocol) or Bubble-CXL. Crosslinking was performed on three pairs of eyes, whereby one eye of each pair was treated with the conventional CXL and the other with the Bubble-CXL method. For Bubble-CXL, the eye’s epithelium was removed, and an intracameral oxygen bubble was injected until 80% of the anterior chamber was filled (Figure 1A). Thereafter, the operation method followed the conventional Dresden protocol [4]. Riboflavin was instilled on the cornea every 5 min for 30 min to diffuse into the stroma (Figure 1B). To induce collagen crosslinking, the cornea was subsequently irradiated with UVA for 30 min, while riboflavin was instilled every 5 min continually (Figure 1C). After the treatment, the oxygen bubble was removed.

**Figure 2:**
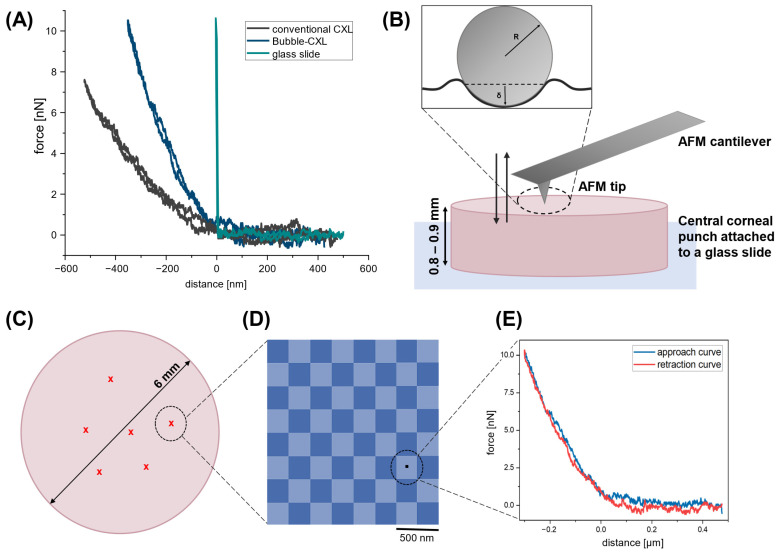
(**A**) Force–distance curves recorded in AFM force spectroscopy: the indentation of the AFM tip into the cornea sample is recorded by the deflection of the AFM cantilever, visible in a non-linear indentation curve. No indentation of the AFM tip was observed on a rigid glass substrate, whereas the indentation depth δ is measurable on softer samples. (**B**) For AFM indentation experiments, the cornea sample was attached to a glass substrate, and force–distance curves were recorded by indenting the parabolic part of the tip into the sample. An elastic half-space is assumed for small indentation forces, and the indentation depth δ depends on the sample stiffness. (**C**) Measurements were performed on different positions on the cornea surface, (**D**) on each position, 8 × 8 force–distance curves were recorded within an area of 4 µm^2^. (**E**) The indentation part of the approach curve (blue, −0.3 µm to 0 µm) was further analyzed for Young’s modulus calculation.

For a stiffness assessment of the prepared cornea samples, atomic force microscopy (AFM) indentation experiments were performed. Deriving elastic properties with AFM by force spectroscopy is a widely used application and allows for characterizing a variety of materials, such as polymer systems [21], collagen [22,23], cells [20,24] or bacterial surfaces [25]. In AFM force spectroscopy, a tip attached to a cantilever, which acts like a Hookian spring, is brought into contact with the sample surface and is subsequently gently pushed into the sample, which results in an upwards deflection of the cantilever. Thereby, the cantilever tip also indents the sample and probes its mechanic and viscoelastic properties. The cantilever is retracted from the surface when a pre-set deflection (force) limit is reached. The deflection of the cantilever versus its movement is recorded and presented in a so-called force–distance curve [26]. Figure 2A shows three force–distance curves recorded during our series of measurements on the corneas of the first pair of eyes. A mostly nonlinear indentation part of such a force–distance curve (Figure 2A, −600 nm to 0 nm) reflects the sample stiffness and, therefore, can be used to derive Young’s modulus [25,26,27]. The AFM tip does not penetrate hard surfaces such as glass substrates, and, therefore, the cantilever deflection curve on glass (Figure 2A, green line) results in a steep indentation curve corresponding to the movement of the cantilever. For softer samples, however, the deflection curve of the force–distance curve is no longer linear (Figure 2A, blue and black line), as the AFM tip indents the surface, with larger indentation depths δ corresponding to lower sample stiffnesses [25,27]. A greater indentation depth and hence a lower stiffness of the cornea treated with conventional CXL was clearly found compared with the stiffness of the cornea treated with Bubble-CXL (Figure 2A).

A schematic illustration of the used experimental setup is shown in Figure 2B. AFM indentation experiments were performed in buffer solution on a corneal punch with a diameter of 6 mm and an average height of 0.87 ± 0.07 mm, attached to a glass substrate. Measurements were performed on various positions of the sample (Figure 2C), whereby on each position the tip was scanned over an 8 × 8 grid on an area of 2 × 2 µm^2^ (Figure 2D). One force–distance curve was recorded on each grid position so that 64 force–distance curves were measured per position (Figure 2E). For quantification, the indentation part of the approach curve of each recorded force–distance curve (Figure 2E, blue curve) was fitted with the Hertz model [18,19] to calculate Young’s modulus, which is an appropriate model for small indentations depths. The indentation of the parabolic part of the AFM tip was approximated by an elastic half-space, as indicated in Figure 2B.

**Figure 3:**
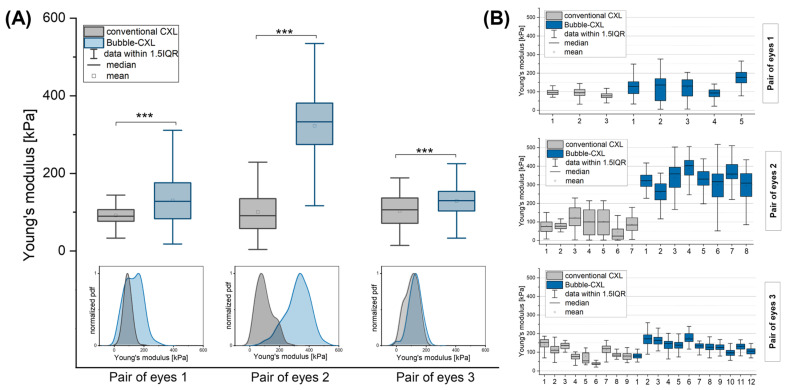
(**A**) Boxplot diagrams of calculated Young’s modulus values of all recorded force–distance curves of corneas treated with conventional CXL versus corneas treated with Bubble-CXL for three pairs of eyes. Corneas treated with Bubble-CXL show a significantly higher stiffness (*p* ≤ 0.001) (indicated with ***) than those treated with conventional CXL. The experimental probability density functions (pdfs) of Young’s moduli show a clear shift to higher Young’s moduli for Bubble-CXL treatment. (**B**) Young’s modulus distributions of force volume measurement (8 × 8 force–distance curves) were recorded at various positions on cornea samples of pairs of eyes 1–3.

Young’s moduli were compared between corneas of the same pair of eyes. For corneas treated with conventional CXL, similar Young’s moduli were found (91.65 ± 22.07 kPa, 100.58 ± 54.78 kPa, 103.31 ± 42.67 kPa (AV ± SD)), whereas corneas treated with Bubble-CXL collectively showed significantly higher Young’s moduli (130.46 ± 59.42 kPa, 322.51 ± 83.61 kPa, 129.06 ± 36.71 kPa (AV ± SD)), when compared to the partner eye treated with conventional CXL (Figure 2C, boxplot diagrams, Table 1). Young’s moduli distributions were constructed from experimental probability density functions (pdfs; equivalents of continuous histograms) (Figure 2C), revealing a shift towards higher Young’s moduli for corneas treated with Bubble-CXL for all pairs of eyes, but most pronounced for pair of eyes 2 with an average increase in stiffness of 220.65%. The stiffness increase was 42.35% for pair of eyes 1 and 24.92% for pair of eyes 3. Our force spectroscopy experiments obviously showed that the enhanced crosslinking effect due to oxygen bubble injection did not always occur homogeneously at all positions of the cornea sample. This is most apparent from the pdfs of pair of eyes 1 (Figure 2C), which contains two prominent peaks in Young’s modulus distribution for the cornea treated with Bubble-CXL. Here, the second peak with higher Young’s moduli values indicates an enhancement of the crosslinking method. In contrast, the first peak overlaps with the peak for conventional CXL, lacking additional crosslinking effects on specific positions of the cornea sample. The variances in Young’s modulus distributions can be traced back to individual positions on which force–volume measurements (8 × 8 force–distance curves per position) were performed (Figure 3B). For pair of eyes 1, no treatment effect for Bubble-CXL was observed for position 4, moderate effects were found on positions 1 to 3, and an apparent effect for position 5. In comparison, this inhomogeneity was less pronounced for pairs of eyes 1 and 2 on different positions of cornea samples treated with conventional CXL. For pair of eyes 3, however, variances between the positions were found for both treatment methods, explaining the overlap of the distributions of all Young’s modulus values (Figure 3A, pdf for pair of eyes 3). Nonetheless, on average, a slight but significant stiffness increase in pair of eyes 3 was found for the cornea treated with Bubble-CXL. 

## 4. Discussion

The efficiency of corneal crosslinking is highly dependent on the environment’s oxygen level. In the treatment method, collagen crosslinking by forming covalent bridges between amino groups of collagen fibrils is induced by singlet oxygen, which leads to increased stiffness and strengthening of the cornea [4]. Diakonis et al. proposed that the oxygen supply from the outer air environment is insufficient for the photochemical reaction during CXL [16] as, after one hour of irradiation with UVA light, more than 65% of riboflavin remained intact [28]. In addition, Kamaev et al. observed total oxygen depletion under a 100 µm cornea flap within 1.5 to 15 s of CXL, depending on UVA irradiance [6]. These findings reveal that oxygen is missing in the stroma after the first seconds of CXL treatment but diffuses back into the stroma until a steady state is reached, at which oxygen transportation through the cornea is matched by its consumption [6,16]. The effect of different oxygen levels in the environment during CXL indicates that a low oxygen environment prevents crosslinking [7]. In contrast, an oxygen-enriched environment increases crosslinking efficiency when performed with conventional CXL (Dresden protocol) [8].

Here, we introduced a novel method for corneal crosslinking (Bubble-CXL), in which oxygen in the eye’s anterior chamber was used for oxygen enrichment in the stroma, thereby serving, together with the outer air environment, as a second oxygen source. By performing AFM indentation experiments, the effect of the treatment method was investigated by comparing Young’s moduli of porcine corneas treated with Bubble-CXL with corneas treated with conventional CXL. Obtained Young’s moduli were in good agreement with findings in other studies, in which cornea stiffness was assessed with AFM [14,15,16]. Young’s modulus of untreated control eyes in these studies was found to be 245.9 ± 209.1 kPa [15] and 14.5 ± 6.0 kPa [16] for the anterior stroma of human eyes and 9.4 ± 1.7 kPa [14] over the entire stroma for porcine eyes. The effect of conventional CXL (Dresden protocol) on cornea stiffness was investigated in these studies, finding a stiffening increase of factor 1.9 to 5.5, resulting in a mean Young’s modulus of 467.8 ± 373.2 kPa [15] and 80.7 ± 44.6 kPa [16] for human corneas and 47.0 ± 23.0 kPa for porcine corneas [14] after stiffening with conventional CXL. In our study, on average, the three porcine eyes, treated with Bubble-CXL showed a significant increase in stiffness compared to those treated with conventional CXL (Dresden protocol), with a stiffening factor of 1.4, 3.2, and 1.2 for pair of eyes 1–3, respectively, thus indicating that intracameral oxygen indeed could lead to higher crosslinking efficiency. CXL is used to treat diseases such as Keratoconus, where the progression of the disease leads to thinning and deformation of the cornea. However, the conventional CXL method only leads to a significant stiffening in the anterior layer of 200 µm of the corneal stroma [29] as the method’s effect is exponentially declining throughout the stroma. The Bubble-CXL method, due to the additional oxygen supply from the anterior chamber, could lead to a higher stiffness in deeper areas of the stroma, resulting in an overall higher Young’s modulus. Furthermore, higher crosslinking efficiency through Bubble-CXL provides the possibility to shorten the treatment time and thus provide higher patient comfort, while still achieving the same corneal stiffening as with the conventional method. 

However, we found that the treatment effect of Bubble-CXL varied for different positions on the cornea sample. We found positions on which almost no stiffness increase was observed, as well as positions with a pronounced effect compared to conventional CXL. This might be an effect of local diffusion speeds of intracameral oxygen into the stroma at various positions within the cornea and/or varying diffusion of riboflavin to deeper corneal layers. In addition, the overall efficiency of Bubble-CXL treatment varied significantly between the three pairs of eyes. For the Bubble-CXL treatment, intracameral oxygen was injected until 80% of the anterior chamber was filled. With this injection method, possible variations in the volume of the anterior chambers between the three pairs of eyes might cause different injection volumes of oxygen applied to the eyes. An approach to better control and achieve a more homogeneous treatment effect could be the standardization of the amount of injected oxygen and controlling the eye pressure during injection.

For further studies, a higher number of eyes needs to be studied to improve the statistical significance. In addition, the short- and long-term safety of adjunctive intracameral oxygen in human eyes needs to be evaluated. There is a risk of intracameral oxygen for the development of cataracts, endophthalmitis, or a translational increase in intraocular eye pressure.

In conclusion, our study indicates that intracameral oxygen might indeed increase the oxygen concentration in the stroma, leading to higher crosslinking efficiency and improvement of the corneal biomechanical properties. Further studies on refining the treatment method to achieve homogeneous stiffening throughout the treated cornea and consistent results within different individuals are aimed for improved standardization of our approach.

## Figures and Tables

**Table 1:** Summary of Young’s moduli (average ± standard deviation) of corneas from three pairs of eyes treated either with conventional CXL or Bubble-CXL. The sample size describes the sum of force–distance curves recorded on various sample positions (number of positions) used for Young’s modulus calculation.

Pair of Eyes	Treatment Protocol	Young’s Modulus [kPa] (AV ± SD)	Number of Positions	Sample Size
1	Conventional CXL (Dresden)	91.65 ± 22.07	3	165
	Bubble-CXL	130.46 ± 59.42	5	220
2	Conventional CXL (Dresden)	100.58 ± 54.78	7	300
	Bubble-CXL	322.51 ± 83.61	8	509
3	Conventional CXL (Dresden)	103.31 ± 42.67	9	409
	Bubble-CXL	129.06 ± 36.71	12	757

## Data Availability

Data presented in this article are available on request from the corresponding authors.
